# Effect of Sodium Hydroxide Concentration in Synthesizing Zinc Selenide/Graphene Oxide Composite via Microwave-Assisted Hydrothermal Method

**DOI:** 10.3390/ma12142295

**Published:** 2019-07-18

**Authors:** Han Kee Lee, Zainal Abidin Talib, Md Shuhazlly Mamat @ Mat Nazira, EnZe Wang, Hong Ngee Lim, Mohd Adzir Mahdi, Eng Khoon Ng, Norita Mohd Yusoff, Batool Eneaze AL-Jumaili, Josephine Ying Chyi Liew

**Affiliations:** 1 Department of Physics, Faculty of Science, Universiti Putra Malaysia, Serdang 43400 UPM, Selangor, Malaysia; 2 Institute of Advanced Technology, Universiti Putra Malaysia, Serdang 43400 UPM, Selangor, Malaysia; 3 Department of Chemistry, Faculty of Science, Universiti Putra Malaysia, Serdang 43400 UPM, Selangor, Malaysia; 4 Wireless and Photonics Network Research Centre, Faculty of Engineering, University Putra Malaysia, Serdang 43400 UPM, Selangor, Malaysia; 5 College of Applied Science, Department of Medical Physics, University of Fallujah, Fallujah 31002, Iraq

**Keywords:** Microwave-assisted hydrothermal method, NaOH concentration, ZnSe/GO composite, Optical band gap

## Abstract

The effect of NaOH solution on the formation of nanoparticles has been the subject of ongoing debate in selenium-based material research. In this project, the robust correlation between the mechanistic growth of zinc selenide/graphene oxide (ZnSe/GO) composite and the concentration of NaOH are elucidated. The ZnSe/GO composite was synthesized via microwave-assisted hydrothermal method and the concentrations of NaOH are controlled at 2 M, 3 M, 4 M, 5 M and 6 M. The XRD spectra show that the crystal phases of the samples exhibited a 100% purity of ZnSe when the concentration of sodium hydroxide (NaOH) was set at 4 M. The further increase of NaOH concentration leads to the formation of impurities. This result reflects the essential role of hydroxyl ions in modifying the purity state of ZnSe/GO composite. The optical band gap energy of ZnSe/GO composite also decreased from 2.68 eV to 2.64 eV when the concentration of NaOH increased from 2 M to 4 M. Therefore, it can be concluded that the optimum concentration of NaOH used in synthesizing ZnSe/GO composite is 4 M. This project provides an alternative green method in the formation of a high purity ZnSe/GO composite.

## 1. Introduction

Chalcogenide semiconductor nanoparticles have optical properties which are widely used in optoelectronic applications [1]. Zinc selenide (ZnSe) is a wide band gap semiconductor with 2.70 eV at room temperature and good optical transmission performance under light. It is a common material used in optoelectronic applications such asphotovoltaic devices, light-emitting devices, transmission windows, and storage devices, as well as in Lasik surgery [2,3]. ZnSe can be synthesized using various methods such as the sol-gel, thermal evaporation, wet chemical synthesis, hydrothermal and solvothermal methods [4,5,6,7,8]. The shape and size of ZnSe are easily controlled using the hydrothermal or solvothermal method. Zhang et al. have reported that the enhancement of emission performance and photocatalytic activity can be achieved by using a ZnSe microsphere with a size of 2 μm [9]. Ashari et al. also successfully reduced the size to 8.9 nm and tuned the optical band gap energy of ZnSe quantum dots to 3.68 eV using the hydrothermal method [10]. Generally, the size of a ZnSe particle can be varied from micro-size to quantum dots [11]. Hence, the uniqueness of the optical and electronic properties of ZnSe can be obtained by controlling its dimension (shape and size) [9,10,11]. Graphene is known as a unique material with its peculiar physical properties, particularly in mechanical, optical and electrical areas. The attention of research on the synthesis and integration of graphene-based composite with others nanomaterials is resulting in the eventual wide application of graphene sheet based devices [9]. Zou et al. presented that the utilization of graphene templates during ZnO growth can improve the field emission, gas sensor and photocatalytic properties of the composite materials [12]. El-Shafai et al. have synthesized graphene oxide/iron oxide and graphene oxide/iron oxide/zirconium oxide nanocomposite. These nanocomposites had high photocatalytic activity. It is proved that the nanocomposite has higher adsorption of Rhodamine B over the surface of GO [13].

Microwave technology has been developed dynamically in recent years. It has been used in the synthesis of materials and various applications [14,15,16] such as food processing, analytical chemistry, heating and the vulcanization of rubber. Umer et al. have reported that pure mordenite zeolite can be synthesized via a microwave approach [17]. Besides, iron-modified activated carbon fiber can also be fabricated by microwave-assisted heating treatment from simulated wastewater [18]. There are numerous articles studying the synthesis and structural modification of zinc oxide nanostructures using the microwave-assisted hydrothermal method. For example, Hasanpoor et al. have reported a flower-type, needle-like and sphere-like nanoparticle via this approach [19]. From previous research, it can be summarized that microwave-assisted synthesis is an excellent method to produce a homogenous sample in a shorter reaction growth time.

There is limited research on the formation of graphene-based ZnSe materials using the microwave synthesis approach. Therefore, the idea of incorporating graphene in the ZnSe crystal matrix is investigated in this project. By mixing graphene oxide with a ZnSe nanoparticle, it can be observed that the graphene oxide layer can reduce the recombination of electron-electron hole pairs during the photon absorption process. It is believed that the lifetime of the photogenerated carriers in samples also improved along with their potential use in optoelectronic applications [20,21]. In addition, there is a lot of research that has reported on synthesizing a chalcogenide semiconductor by varying parameters such as the types of precursors, temperature, reaction time, types of surfactants and the concentration of surfactants. Typically, the size of the semiconductor can be controlled by varying the concentration of hydroxyl ions [22,23,24].

However, the research related to the effect of NaOH concentration on synthesizing ZnSe materials is yet to be fully explored. Ben Nasr et al. have reported the effect of the concentration of hydroxyl ions on the properties of ZnS thin films [25]. Meanwhile, Farjami Shayesteh et al. have reported the effect of concentration of hydroxyl ions on the structural and optical properties of ZnS nanoparticles [1]. It is found that the solution hydroxyl ions could improve the optical properties of the ZnS by modifying the optical band gap energy of the materials. Thus, the objective in the present work is to study the effect of NaOH concentration on synthesizing ZnSe/GO composite via the microwave-assisted hydrothermal method. The effect of NaOH concentration on the optical properties of the ZnSe/GO composite will be discussed. The effect of hydroxyl ions on the reaction mechanism will be elaborated in detail.

## 2. Materials and Methods

The chemicals used were sodium hydroxide (purity ≥ 98%, Fisher Scientific, Hampton, NH, United State), hydrazine hydrate (Fisher Scientific), diethanolamine (purity ≥ 99.5%, Merck, Darmstadt, Germany) selenium (purity ≥ 99%, HmbG Chemical, Hamburg, Germany), and zinc chloride (purity ≥ 97%, HmbG Chemical, Germany). A graphene oxide sheet was purchased from GO Advanced Solutions Sdn. Bhd.

The previously reported method was used in this study [20]. In detail, a graphene oxide solution was prepared by dispersing (2.5 ± 0.1) mg of graphene in 25 mL of deionized water. Sodium hydroxide with different molarity, which were 2 M, 3 M, 4 M, 5 M and 6 M, were prepared. Subsequently, a 5 mL of hydrazine hydrate, 0.1974 g of selenium powder and 2 mL of diethanolamine were then added into the solution and stirred for 5 min. A deep-red solution was formed in this process. Then, (0.2726 ± 0.010) g of zinc chloride was added into the mixture and stirred for 2 h. The solution was then poured into an autoclave chamber (Parr Microwave Acid Digestion Vessels 4782, Parr Instrument, Moline, IL, United State) and heated in a microwave oven (Electrolux Microwave Oven ELE-EMM 2001S, Electrolux, Kuala Lumpur, Malaysia) for 3 min under the power irradiation of 700 W. Later, the sample was rinsed with a flow of ethanol and deionized water 5 times. Finally, the sample was dried in a vacuum chamber at 60 °C for 24 h to obtain a powder sample. The structural properties of the samples were characterized using X-ray diffraction. The optical properties of the sample were measured using Raman spectroscopy and UV-Visible spectroscopy. Meanwhile the morphology of the sample was evaluated using a field emission scanning electron microscope (FESEM). Specifically, the X-ray diffraction patterns were recorded using Panalytical’s (Philips, PW3040/60, Malvern Panalytical Ltd., Royston, UK) with CuKα radiation generated at 40 kV and 40 mA at 20° to 80°. Raman spectra were obtained using WITec Alpha 300R (WITec, Knoxville, TN, United State) with an excitation laser source of 488 nm. UV-Vis spectroscopy (Shimadzu-UV3600, Shimadzu, Selangor, Malaysia) was used to determine the optical properties of the sample. The morphology of the sample was determined using FESEM (Hitachi SU8000, Hitachi, Krefeld, Germany) with an accelerating voltage of 3 kV and a working distance of 3 nm.

## 3. Results

In this session, the experimental results, such as phase and structural, optical, and morphological results, are presented.

### 3.1. Phase and Structural Results

**Figure 1:**
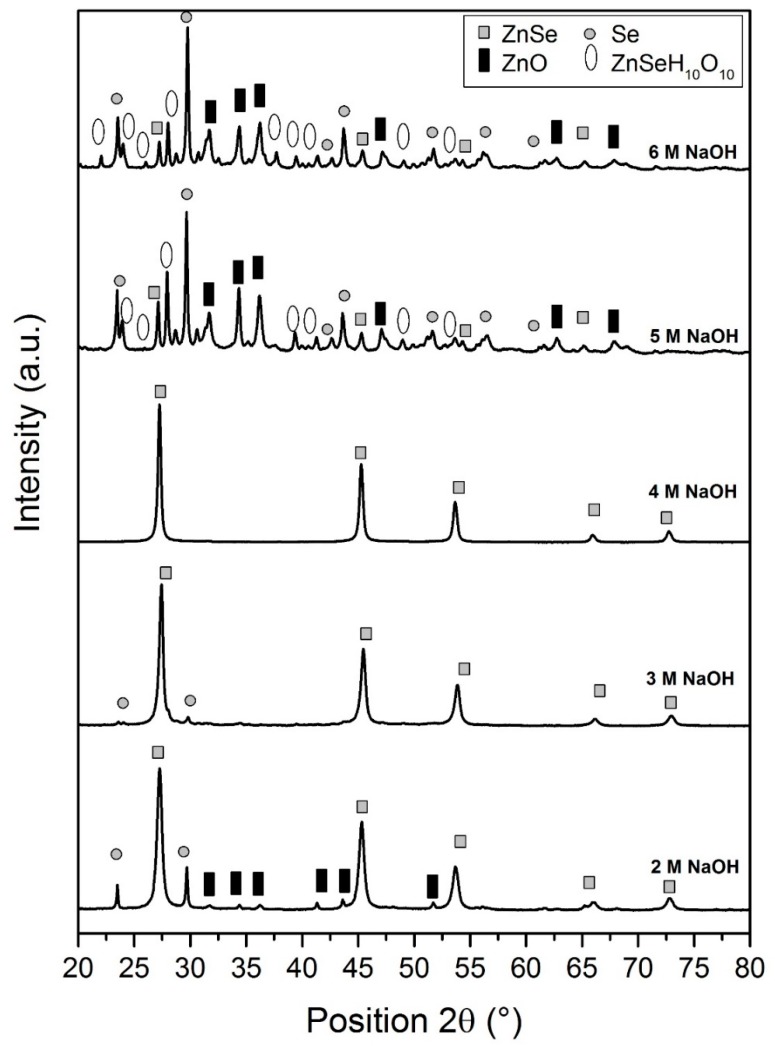
XRD spectra of the zinc selenide/graphene oxide (ZnSe/GO) composite by varying the sodium hydroxide (NaOH) concentration.

Figure 1 shows the XRD spectra for the sample synthesized in different concentrations of NaOH. The solution pH was measured before heating process and recorded as 11.17, 11.38, 11.47, 11.53 and 11.89 when the concentration of NaOH is 2 M, 3 M, 4 M, 5 M and 6 M, respectively. The solution pH was measured using a pH meter (Mettler Toledo –Seven Easy, Mettler-Toledo, Greifensee, Switzerland) before the suspension was heated. The powder sample was placed in a sample holder and then the XRD analaysis was carried out. Table 1 shows the composition of the sample obtained from different concentrations of NaOH.

### 3.2. Optical Results

**Figure 2:**
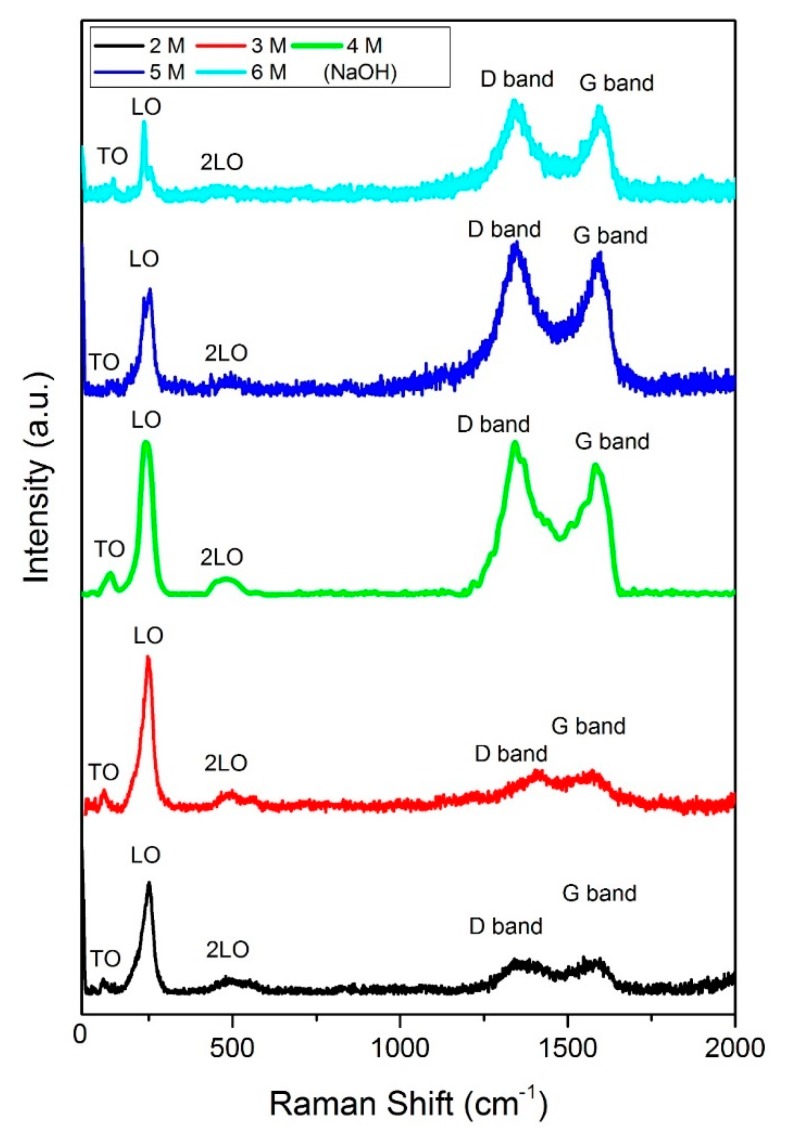
Raman spectra of ZnSe/GO composite synthesized with different NaOH concentrations.

**Figure 3:**
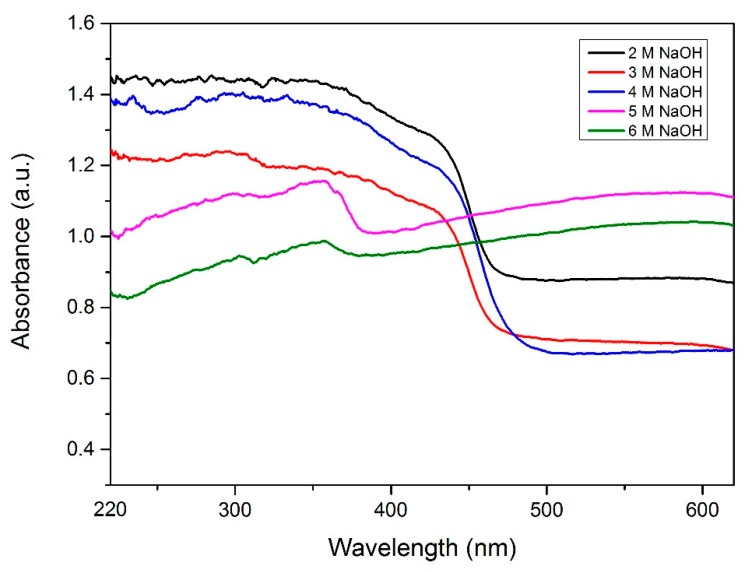
UV-Vis absorption spectra for the sample synthesized with different concentrations of NaOH.

**Figure 4:**
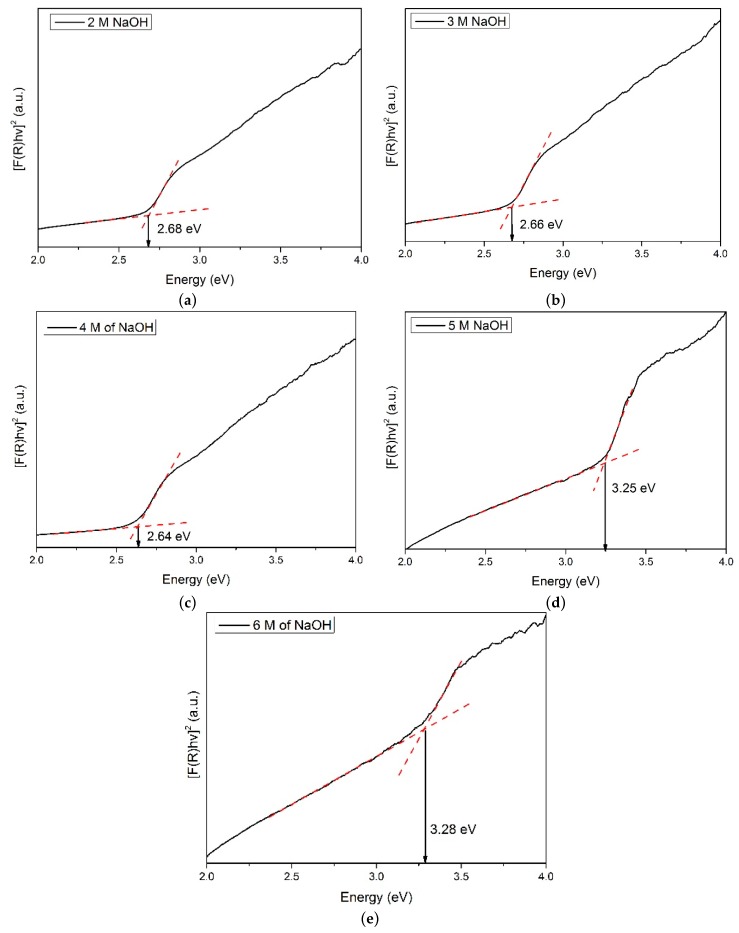
Kulbeka–Munk approach for samples synthesized with (**a**) 2 M, (**b**) 3 M, (**c**) 4 M, (**d**) 5 M, and (**e**) 6 M of NaOH.

Figure 2 shows the Raman spectra for all the samples synthesized with different concentrations of NaOH. It can be observed that a ZnSe/GO composite is formed as proved from the functional peaks of ZnSe and GO. Figure 3 shows the UV-Vis absorbance spectra for all the samples prepared with various NaOH concentrations. Figure 4 shows a plot of [F(R). hυ]^2^ against energy for all the samples. The data was analysed using the Kubelka–Munk approach. The Kubelka–Munk equation is stated as follows:

[F(R)·hυ]^2^*=* β(hυ –E_g_) (1)

where F(R) is the Kulbeka–Munk function, hυ is the photon energy, β is constant and E_g_ is the optical band gap energy. From the graph, the optical band gap energy was obtained by extrapolating the linear part of the plot to the baseline.

### 3.3. Morphological Results

**Figure 5:**
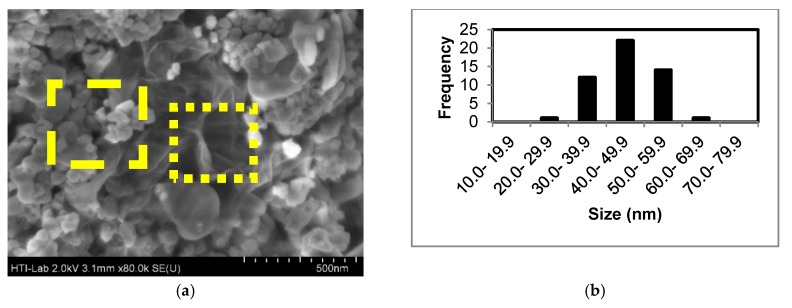
(**a**) FESEM image and (**b**) size distribution graph of ZnSe/GO composite synthesized with 4 M of NaOH.

Figure 5 shows FESEM images of the sample synthesized using 4 M of NaOH. The average particle size of the sample was calculated. Furthermore, the particle size distribution graph of the sample was plotted.

## 4. Discussion

From the XRD results, it is confirmed that the concentration of NaOH affects the formation of ZnSe. The samples synthesized from 2 M, 3M and 4 M of NaOH exhibited cubic ZnSe (ICSD 98-009-1252) peaks at position 2θ of 27.46°, 45.45°, 53.89°, 66.17° and 72.97°, with crystal planes of (111), (022), (113), (004), and (133), respectively. Small and minor ZnSe peaks were observed in the sample synthesized from 5 M and 6 M of NaOH. For samples synthesized in 2 M and 3 M of NaOH solution, there is a ZnSe phase with secondary phases such as selenium (ICSD 98-001-2230) and zinc oxide (ICSD 98-004-0986). The purity of the ZnSe also increased from 88.2% to 100.0% when the concentration of NaOH increased from 2 M to 4 M. Meanwhile, the percentage of ZnSe decreased to 27.7% and 6.0% when the concentration of NaOH increased to 5 M and 6 M. Secondary phases such as selenium, zinc oxide and zinc bis(hydrogenselenate (IV)) tetrahydrate (Zn(HSeO_3_)2(H_2_O)_4_) (ICSD 98-003-3368) were detected. Moreover, the crystallite size of the sample was also calculated using Scherrer’s equation [20]:

L= Kλ/(βcosθ)(2)

where L is the crystallite size, K is a Scherrer constant (K = 0.89), λ is X-ray wavelength, β is full-width half maximum of the peak and θ is the Bragg’s angle. The crystallite size was calculated with respect to the dominant (111) plane. Based on the calculation, the crystallite size of the sample increased when the concentration increased from 2 M to 6 M of NaOH, and the sizes are 21.0 nm, 31.5 nm, 36.0 nm, 50.3 nm, and 52.0 nm, respectively. The NaOH solution has a particular impact on the dispersity, which leads to the coalescence of the ZnSe nanoparticles and indirectly increases the particle size [26].

Raman spectroscopy was used to identify the graphene oxide phase in the sample. Figure 2 shows the Raman spectra for all the samples synthesized using different concentrations of NaOH. Five peaks were observed at wavenumbers of ~115.4 cm^−1^, ~244.5 cm^−1^, ~499.7 cm^−1^, ~1351.2 cm^−1^ and ~1592.6 cm^−1^, which signified the formation of a ZnSe/GO composite. The peaks at ~115.4, ~244.5 and ~499.7 cm^−1^ are indexed to the transverse optical (TO), longitudinal optical (LO) and second order longitudinal optical (2LO) mode of the ZnSe compound [27,28]. On the other hand, at higher wavelengths, two peaks are observed around ~1351.2 (D band of graphene oxide) and ~1592.6 cm^−1^ (G band of graphene oxide). Similar to the previous session, the result is in agreement with the data reported by Lee et al., 2017 who synthesized a cactus-like ZnSe/GO composite [20]. Therefore, the proposed reaction mechanism of the formation of ZnSe, which is similar to Feng et al. [29] is described as below:

N_2_H_4_ + H_2_O ↔ N_2_H_5_^+^ + OH^− ^ (3)

>

N_2_H_5_^+^ + 2Se + 5OH^−^ → N_2_ + 2Se^2−^ + 5H_2_O (4)

ZnCl_2_ → Zn^2+^ + 2Cl^−^ (5)

Zn^2+^ + 2OH^−^ → Zn(OH)_2_ (6)

Zn(OH)_2_ + 2OH^−^ → Zn(OH)_4_^2−^ (7)

Zn(OH)_4_^2−^ + DEA → [Zn(OH)_4_^2−^DEA] (8)

[Zn(OH)_4_^2−^DEA] + Se^2−^ → ZnSe + 4OH^−^ + DEA (9)

When the concentration of NaOH is below 4 M, the growth of zinc oxide nanoparticles is favorable. In low concentrations of hydroxyl ion (OH^−^), it is believed that the primary ion of Zn(OH)_4_^2^^−^ cannot be formed and this results in the formation of Zn(OH)_2_. The unstable state of zinc hydroxide is an unstable compound, which will eventually reduce to a stable form of the zinc oxide nanoparticle, as in Equation (10) [30].

Zn(OH)_2_ → ZnO + 2H_2_O (10)

On the other hand, if there are too many hydroxyl ions, it will react with selenium to form SeO_3_^2^^−^ ions [31]. Thus, it leads to the formation of Zn(HSeO_3_)_2_.4H_2_O (zinc bis(hydrogenselenate(IV)) tetrahydrate). The proposed mechanism is described below:

3Se + 6OH^−^ → 2Se^2−^ + SeO_3_^2−^ + 3H_2_O (11)

SeO_3_^2−^ + H^+^ → HSeO_3_^−^ (12)

[Zn(OH)_4_^2−^ DEA] + 2 HSeO_3_^−^ → Zn(HSeO_3_)_2_ + DEA + 4 OH^−^ (13)

The UV-Vis absorption spectra of the samples are detected from 220 nm to 620 nm. For the sample synthesized using 2 M, 3 M, and 4 M of NaOH, a strong absorption peak was observed at 480 nm, which represented the absorption of the ZnSe/GO composite. This result is in accordance with the data reported by Lee et al. [24]. For the sample synthesized using 5 M and 6 M of NaOH, an absorption peak was observed at 378 nm, which can be assigned to the direct band edge absorption of zinc oxide [32]. The samples synthesized with 5 M and 6 M of NaOH also exhibit broad emission in the entire range. This occurrence reflected the formation of the ZnO/GO composite with a similar trend to that which is reported by Li et al. and Chen et al. [33,34]. The absorption of all samples is dominant in the UV-Vis region. This is because GO is a good light absorber and leads to a high absorption intensity of 0.6 at the visible region.

The optical band gap energy (E_g_) of the samples is estimated using the Kubelka–Munk approach. A plot of [F(R). hυ]^2^ against energy is presented in Figure 4. The optical band gap energy for samples synthesized using different NaOH concentrations of 2 M, 3 M, 4 M, 5 M and 6 M is recorded as (2.68 ± 0.02) eV, (2.66 ± 0.02) eV, (2.64 ± 0.02) eV, (3.25 ± 0.06) eV, and (3.28 ± 0.04) eV, respectively. There were only slight changes in the optical band gap energy when the concentration of NaOH increased from 2 M to 4 M. The slight changes in the optical band gap energy can be related to the crystallite size effect. However, a large difference in optical band gap energy was observed in the sample synthesized using 5 M and 6 M of NaOH. This observation could be due to the higher percentage of zinc oxide that is formed in the composite material.

From the FESEM image, it can be clearly observed that the sample consists of two phases which are (i) particle (dash-border box); and (ii) wrinkle sheet (dot-border box). The particle size of the sample is around (45.8 ± 1.4) nm. The particle is referred to ZnSe while the wrinkled sheet is assigned to graphene oxide sheet. Thus, it can be concluded that ZnSe/GO composites are successfully synthesized using the microwave-assisted hydrothermal method where ZnSe is grown on top of the GO sheet, and confirmed by the Raman characterization.

## 5. Conclusions

In conclusion, the optimum NaOH concentration used to synthesize high purity ZnSe/GO composite is 4 M. The XRD result shows that the samples possess the highest percentage of ZnSe. The experiment proved that the hydroxyl ions play an essential role in producing high purity ZnSe/GO composite. The optical band gap energy of ZnSe/GO composite is shifted from (2.68 ± 0.02) eV to (2.64 ± 0.02) eV when the concentration of NaOH increased from 2 M to 4 M. The slight change in the band gap is due to the growth of the crystallite size when a higher concentration of NaOH is used. It is confirmed that the concentration of NaOH at 5 M and 6 M is not suitable to be used as a solution for producing high purity ZnSe/GO nanocomposite. This is due to the formation of ZnO and Zn(HSeO_3_)_2_(H_2_O)_4_.

## Figures and Tables

**Table 1:** Phases of the synthesized ZnSe/GO composite by varying the NaOH concentration.

NaOH (M)	Composition (%)			
	ZnSe	Se	ZnO	Zn(HSeO_3_)_2_(H_2_O)_4_
2	88.2	10.2	1.6	-
3	98.2	1.8	-	-
4	100.0	-	-	-
5	27.7	35.6	5.9	30.8
6	6.0	40.0	26.0	28.0
